# Parliament and the Professions

**Published:** 1920-03-27

**Authors:** 


### Parliament and the Professions.

### Indian Medical Service.

In answer to a question to the Secretary of State for India, it was stated that in a report received from the Government of India in July last they estimated that 166 officers were required to bring the Indian Medical Service up to the strength required by present conditions; forty-six officers have been appointed to the Service in the past year.

### R.A.M C. in Mesopotamia

Sir A. Williamson informed Sir W. Cheyne that every endeavour continues to be made to relieve medical officers of the Special Reserve and Territorial Force serving in Mesopotamia who, on account of age, length of service in the East, and personal hardship, are considered by the General Officer Commanding to be eligible for release. The establishments are being reduced to the greatest possible extent, having regard to the care of the sick and the protection of the garrison; and regular Royal Army Medical Corps officers are being sent out as far as the available resources and the heavy demands to be met elsewhere permit.

### Hospitals and the National Relief Fund.

Mr. W. Graham asked the Prime Minister whether his attention had been called to the present financial position of many infirmaries and hospitals in all parts of the country; and whether, having regard to the fact that practically all of them rendered great service in treating the wounded and disabled at low rates of payment, and that their present position is in part due to circumstances arising from the war, he will support an appeal to the authorities of the National Relief Fund that a portion of the unexpended balance of the Fund should be allocated among these institutions, and so endorse the claim which many of them are now making upon the sum at the disposal of the National Relief Fund authorities.

The Prime Minister said that the Government have no responsibility for the administration of the Fund, which was subscribed for the relief of distress due to the war, and it rests with the Committee to determine what objects come within its scope. But he understood that in the opinion of the Committee the Fund cannot properly be applied in making good the deficits in the administration of the general hospitals throughout the country.

### Nursing Services and the Ministry of Health.

Dr. Addison informed Captain Terrell that as the total membership of a Consultative Council is restricted by Order in Council to twenty, he regretted that within this limit it was not found possible to include persons, such as representatives of the county nursing associations and of Queen Victoria's Jubilee Institute for Nurses, in the Council on Medical and Allied Services. He hoped, however, to have the advantage of their assistance on any committee of the Council appointed to consider the future organisation of nursing services.

